# Pain Status and Disability in Activities of Daily Living Among Older Adults in China: Evidence From CHARLS 2020

**DOI:** 10.1155/prm/4974163

**Published:** 2025-07-16

**Authors:** Jingjing Chu, Luxi Weng, Wen Jin, Xi Yin, Qin Xu, Zherong Xu

**Affiliations:** ^1^Department of Medical Records, The First Affiliated Hospital, Zhejiang University School of Medicine, Hangzhou, China; ^2^Department of Stomatology, The First Affiliated Hospital, Zhejiang University School of Medicine, Hangzhou, China; ^3^Department of Nutrition, The First Affiliated Hospital, Zhejiang University School of Medicine, Hangzhou, China; ^4^Department of Geriatrics, The First Affiliated Hospital, Zhejiang University School of Medicine, Hangzhou, China

**Keywords:** BADL, disability, IADL, older adults, pain status

## Abstract

**Background:** Pain status is a common concern among older adults and has been linked to functional limitations. This study aimed to examine the association between pain status and disabilities risk in basic activities of daily living (BADL) and instrumental activities of daily living (IADL) in older adults in China, using data from the 2020 China Health and Retirement Longitudinal Study (CHARLS).

**Methods:** A cross-sectional analysis was conducted using data from 8102 participants aged 60 and older from the 2020 CHARLS. Univariate and multivariate binary logistic regression analyses were performed to assess the association between pain status and BADL/IADL disabilities. We further examined the contribution of each covariate and categorized participants by pain location and number of pain sites. Subgroup analyses were conducted to examine the consistency of findings across demographic and health-related factors.

**Results:** Pain status was significantly associated with higher odds of both BADL and IADL disabilities (*p* < 0.05), even after adjusting for covariates. Self-rated health and depressive symptoms exerted the greatest influence on the OR values. Pain in any anatomical region, particularly when present at multiple sites, was associated with increased odds of disability. Head and neck pain was specifically associated with IADL disability, while pain in the upper limbs, torso, and lower limbs was associated with both BADL and IADL disabilities. Subgroup analyses confirmed the robustness of these associations.

**Conclusions:** Pain status, especially multisite pain, is significantly associated with BADL and IADL disabilities in older Chinese adults. Although causality cannot be inferred due to the study's cross-sectional design, these findings underscore the importance of addressing pain alongside other health and psychological factors when developing strategies to support functional independence in aging populations.

## 1. Introduction

Pain status is a prevalent and persistent health issue among older adults, significantly affecting their quality of life and functional independence [1]. Pain status is not immediately life-threatening. Individuals often continue to live with their pain, and hence these conditions are common in both developed and developing countries [2, 3]. As populations worldwide continue to age, there is a growing need to understand how pain status influences specific aspects of daily living in older adults. Basic activities of daily living (BADL) and instrumental activities of daily living (IADL) are well-established indicators for evaluating disability in this population. BADL refers to fundamental self-care tasks, while IADL encompasses more complex activities needed for independent community living. Pain status, by limiting mobility, strength, or motivation, may significantly increase the likelihood of disability as reflected in these measures. Understanding how pain status relates to BADL and IADL is therefore critical for identifying individuals at risk and developing targeted interventions to maintain or improve their functional independence.

Extensive research indicates that pain status negatively impacts physical function, cognitive performance, and emotional health in older adults [4, 5]. Pain status often intersects with cognitive function and emotional well-being [6, 7], complicating its assessment and management in older populations. Most previous studies have not accounted for the number or specific sites of pain status or adjusted for important confounders like physical capacity, depressive symptoms, or cognitive status [8, 9]. In China, where rapid population aging is underway, understanding the relationship between pain status and BADL/IADL disabilities is particularly pressing. The China Health and Retirement Longitudinal Study (CHARLS) provides a comprehensive and nationally representative dataset that allows for an in-depth examination of these associations in older Chinese adults.

This study aims to explore how pain status contributes to BADL and IADL disabilities in older adults in China while also examining the influence of covariates. Using key variables extracted from the CHARLS 2020 dataset, we defined a well-characterized study population by applying strict exclusion criteria. A multivariate modeling approach was employed to quantify the association between pain status and the risk of BADL and IADL disabilities while also assessing the contributions of key covariates. Participants were further categorized by pain location and the number of pain sites, and subgroup analyses were conducted to validate the robustness of the findings across various demographic and health-related profiles. Through this approach, our research is expected to contribute to a deeper understanding of how pain status affects disability in activities of daily living in older adults.

## 2. Methods

### 2.1. Study Population

This study was a cross-sectional analysis based on data from the 2020 wave of the CHARLS, a nationally representative survey of Chinese adults aged 45 years and older. CHARLS was initiated in 2011 by the National School of Development at Peking University, with an initial baseline sample of 17,708 individuals from 10,257 households, selected using a stratified, multistage probability proportional to size (PPS) sampling method across 28 provinces. In subsequent survey waves, the cohort experienced attrition due to mortality and loss to follow-up. To address this and maintain representativeness, the project team conducted limited sample refreshment in certain regions during specific years. As a result, the total number of respondents increased over time, reaching 19,395 in the 2020 wave. Data collection for this wave was primarily conducted in 2020, with delays in some regions due to the COVID-19 pandemic. The final dataset for the 2020 wave was officially released on the CHARLS website on November 16, 2023. Detailed survey methodologies have been published previously [10]. The CHARLS survey received ethical approval from the Peking University Bioethics Committee (IRB No. IRB00001052-11014), and all respondents provided informed consent.

**Figure 1:**
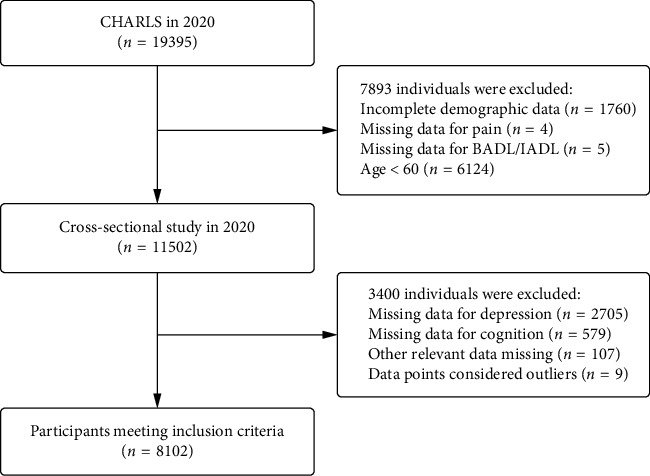
Flow diagram for participants selection.

The current analysis focused on individuals aged 60 years or older from the most recent CHARLS data in 2020. After starting with 19,395 participants, we applied the following exclusion criteria: (1) age < 60 years; (2) incomplete demographic data; (3) missing BADL and/or IADL data; (4) incomplete pain data; (5) missing critical variables (such as those on depression and cognition); and (6) data points identified as outliers, including implausible values such as age greater than 120 years or categorical variables recorded outside their predefined coding ranges. The final sample size of 8102 participants was determined based on the availability of data from the CHARLS 2020 wave after applying the exclusion criteria. The large sample size and variability in participant characteristics ensured sufficient statistical power to detect significant associations and provide robust estimates. The flow diagram illustrating the participant selection process is shown in Figure 1. The final dataset encompassed detailed information on demographics, socioeconomic status, and health. This comprehensive dataset was utilized to investigate the associations between pain status and disabilities in daily living activities while also examining the impact of various health-related and socioeconomic factors on these relationships.

### 2.2. Assessment of Functional Ability

Functional ability was assessed using standardized measures for BADL and IADL [11, 12]. BADL assessed the ability to perform basic self-care tasks such as dressing, bathing, eating, getting out of bed, toileting, and managing urination and bowel movements. IADL evaluated more complex tasks required for independent living, including household chores, cooking, shopping, telephone use, financial management, and medication adherence.

Responses to each item were categorized into four levels: (1) No difficulty, (2) difficulty but can still perform the task, (3) difficulty and need assistance, and (4) unable to perform the task. Participants who reported any level of difficulty, ranging from (2) “Difficulty but can still perform the task” to (4) “Unable to perform the task,” in any BADL or IADL item were classified as having a BADL or IADL disability, respectively [13].

### 2.3. Assessment of Pain

Participants were categorized into two groups based on whether they reported experiencing any pain-related discomfort. Those reporting no pain served as the reference group. For participants who reported pain, detailed anatomical sites were documented based on their responses to the CHARLS questionnaire, including the head, neck, shoulder, arm, wrist, fingers, chest, stomach, back, waist, buttocks, leg, knees, ankle, toes, and other specified areas. Additionally, these pain sites were further grouped into four anatomical regions: the head and neck region (including head and neck), the upper limbs (shoulder, arm, wrist, and fingers), the torso (chest, stomach, back, waist, and buttocks), and the lower limbs (leg, knees, ankle, and toes). This classification allowed for the assessment of associations between pain distribution and functional disability outcomes. Participants with pain were further classified into three categories based on the number of reported pain sites: no pain, single-site pain, and multisite pain. To comprehensively capture the relationship between pain and disability in BADL and IADL, the associations were examined from several perspectives: (1) comparing those with no pain, single-site pain, and multisite pain; (2) comparing those with pain in different anatomical regions; and (3) adjusting for key covariates. This approach allowed the investigation of whether the effect of pain status on disability varied by specific body regions and number of sites.

### 2.4. Potential Covariates

Depressive symptoms were assessed using the 10-item Center for Epidemiologic Studies Depression Scale (CES-D) [14]. The CES-D has been validated for Chinese middle-aged and elderly populations [15]. Scores ranged from 0 to 30, with higher scores indicating more severe depressive symptoms. A score ≥ 10 was defined as indicative of depression [16].

Cognitive function was assessed using a modified version of the Telephone Interview for Cognitive Status (TICS) questionnaire [17]. The overall cognitive score was calculated by summing the scores from four domains: orientation (5 points), computation (5 points), memory (20 points), and drawing (1 point), with a total possible score of 31 points [18]. Higher scores indicate better cognitive performance. Physical activity (PA) levels were assessed using a modified International Physical Activity Questionnaire Short Form (IPAQ-SF) [19, 20]. Activities were categorized by intensity, and frequency (days/week) and duration (minutes/day) were recorded. Participants were subsequently classified into light, moderate, or intensive PA groups.

Other potential covariates were chosen based on previous studies and grouped into two main categories [18, 21, 22]. The first included social and lifestyle factors: age, gender, education, marital status, living area, and alcohol and tobacco use. The second category addressed history of falls, self-report health status, life satisfaction and number of comorbidities (hypertension, dyslipidemia, diabetes, cancer, stroke, heart disease, lung disease, liver disease, kidney disease, digestive disease, mental health disorders, memory disorders, asthma, arthritis).

### 2.5. Statistical Analysis

Continuous variables were presented as mean ± standard deviation (SD). For comparisons between groups, the *t*-test or analysis of variance (ANOVA) was applied to normally distributed continuous data, while the Wilcoxon rank-sum test or Kruskal–Wallis test was used for non-normally distributed data. Categorical variables were expressed as frequencies or percentages and were analyzed using the chi-square test.

To evaluate the association between pain status and disability outcomes for BADL and IADL, univariable and multivariable binary logistic regression analyses were performed. Univariable analyses were initially conducted to identify covariates significantly associated with BADL and IADL disabilities. Multivariable models included covariates selected based on clinical relevance and statistical significance in univariable analyses. To assess multicollinearity among the covariates included in the multivariable models, variance inflation factors (VIFs) were calculated. For categorical variables with more than two levels, generalized VIFs were adjusted. Results were reported as odds ratios (ORs) with corresponding 95% confidence intervals (CIs). Subgroup analyses were performed to evaluate the robustness of results across demographic and health-related strata. Efforts were made to minimize bias throughout the study. Selection bias was addressed by including only participants with complete and reliable data. To control for confounding bias, relevant covariates were included in the multivariate analysis to adjust for their potential influence on the outcomes of interest.

The contribution of each covariate to the overall effect size was assessed using the “Chest” package in R software. All statistical analyses were performed using R software (version 4.3.2), and a two-sided *p*-value < 0.05 was considered statistically significant.

## 3. Results

### 3.1. Baseline Characteristics of Participants

Table 1 presents the demographic and baseline characteristics of participants stratified by pain status. There were no significant differences between the pain and nonpain groups in terms of age, smoking status, or physical activity levels (*p* > 0.05). However, several characteristics differed significantly between groups (*p* < 0.001).

Participants in the pain group were more likely to be female (57.05% vs. 39.34%), reside in urban areas (67.77% vs. 62.91%), have lower levels of education, and report lower levels of life satisfaction. They also reported poorer self-rated health (37.63% vs. 11.88%) and a higher prevalence of depressive symptoms (46.50% vs. 21.48%). Clinically, individuals with pain had higher rates of comorbidities, falls, and functional disability (*p* < 0.001). BADL disability was present in 35.68% of participants with pain, compared to 11.31% of those without pain. Similarly, IADL disability was observed in 35.83% of the pain group versus 13.85% in the nonpain group. In addition, the cognitive score in the pain group was lower than in the nonpain group (*p*=0.003). These findings highlighted significant differences in sociodemographic and health characteristics between individuals with and without pain.

### 3.2. Multivariate Logistic Regression Analysis of BADL and IADL Disabilities

Using BADL and IADL disabilities as dependent variables, and the presence of pain status as the independent variable, stepwise logistic regression identified 10 significant covariates for inclusion in the final model. Participants with pain had a higher risk of disability compared to those without pain. Specifically, the adjusted OR for BADL disability was 2.34 (95% CI: 2.04–2.69), and the adjusted OR for IADL disability was 1.91 (95% CI: 1.67–2.19).

Further analysis revealed that older age, depression, history of falls, poor self-rated health, and lower life satisfaction were all associated with an increased risk of both BADL and IADL disabilities. Conversely, being male and having higher cognitive scores were associated with a lower risk of disability (*p* < 0.05). Education level also showed a significant inverse association with disability risk. Compared to participants with no education, those with higher educational attainment were less likely to experience BADL or IADL disabilities. Specifically, individuals with a high school education or above had the lowest odds of disability (OR = 0.66 for BADL and OR = 0.63 for IADL; both *p* < 0.001). Having two or more chronic conditions elevated the risk of BADL disability [OR = 1.35, 95% CI: 1.15–1.58] and IADL disability [OR = 1.30, 95% CI: 1.11–1.52] compared to those without chronic diseases. In contrast, having only one chronic condition did not show a statistically significant increase in disability risk. Regarding physical activity, using light physical activity as the reference, moderate and intensive levels did not significantly reduce the risk of BADL disability (*p* > 0.05). However, both moderate (*p*=0.011) and intensive (*p*=0.003) physical activities were associated with a significantly reduced risk of IADL disability. To ensure the stability of the regression estimates, multicollinearity was assessed among the covariates included in the multivariate models. Since the same set of predictors was used for both BADL and IADL models, VIFs were calculated once. All VIF values were below 2, indicating no significant multicollinearity among the predictors. These findings are summarized in Table 2.

### 3.3. Contribution of Each Covariate to the Effect Size

**Figure 2:**
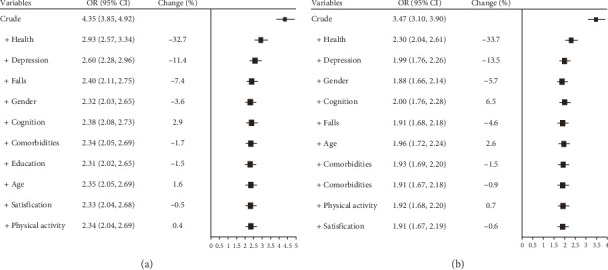
Impact of covariates on the association between pain status and disability risk in BADL (a) and IADL (b).

Figure 2 illustrates the changes in OR values for BADL (Figure 2(a)) and IADL (Figure 2(b)) disabilities after adjusting for covariates. In univariate analysis, the OR for BADL disability with pain status was 4.35 (95% CI: 3.85–4.92), and the OR for IADL disability was 3.47 (95% CI: 3.10–3.90). After adjusting for 10 covariates in multivariate logistic regression, the risks were 2.34 (95% CI: 2.04–2.69) for BADL and 1.91 (95% CI: 1.67–2.19) for IADL, confirming that pain status remained an independent risk factor.

Further analysis was conducted to examine the contribution of each covariate to the effect size within the multivariate logistic regression model. Among all covariates, self-rated health exerted the greatest influence on the OR values, followed by depression. History of falls had a more pronounced effect on BADL disability, while gender and cognitive function had more substantial impacts on IADL disability. Life satisfaction and physical activity intensity had relatively smaller effects on the OR values.

### 3.4. Association of Pain Location and Number of Pain Sites With Disability

Pain location was categorized into head and neck, upper limb, torso, and lower limb. Unadjusted analyses indicated that pain in all four regions was significantly associated with BADL and IADL disabilities (*p* < 0.001). After adjusting for 10 covariates, head and neck pain was no longer significantly associated with BADL disability (*p*=0.965) but remained a significant risk factor for IADL disability (*p*=0.006). Pain in the upper limb, torso, and lower limb regions remained significantly associated with both BADL and IADL disabilities (*p* < 0.05).

Participants were also categorized based on the number of pain sites: no pain, single-site pain, and multisite pain. Compared to those without pain, both single-site and multisite pain were associated with an increased risk of BADL and IADL disabilities (*p* < 0.05). Moreover, the magnitude of association was greater for multisite pain than for single-site pain (Table 3).

### 3.5. Subgroup Analysis of Disability Risk Associated With Pain

**Figure 3:**
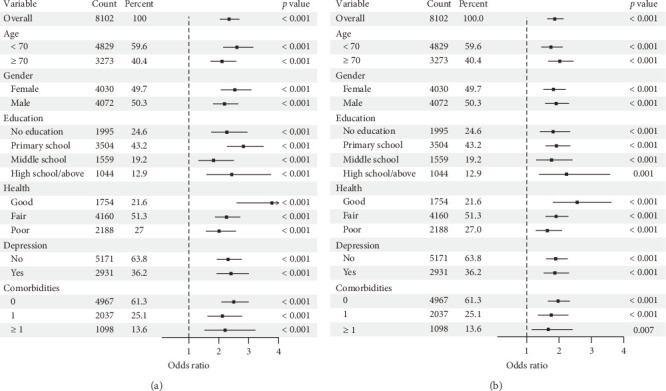
Forest plots of subgroup analyses for pain-related disability risk. (a) Subgroup analysis of BADL; (b) subgroup analysis of IADL.

Subgroup analyses were performed to examine the association between pain status and disability across demographic and health-related strata, including age, gender, education, self-reported health, depression, and comorbidities. Pain status consistently emerged as a significant risk factor for both BADL and IADL disabilities.  Figure 3(a) presents the forest plot for BADL disability risk, while Figure 3(b) displays the corresponding results for IADL, demonstrating consistent associations across all subgroups. The complete odds ratios with 95% confidence intervals are provided in Table 4, which served as a quantitative reference to complement the graphical results in Figure 3.

## 4. Discussion

Pain status and the resulting level of disability represent a global healthcare crisis of epidemic proportions [23]. This study demonstrated that pain status significantly increases the risk of both BADL and IADL disabilities among older adults in China, even after adjusting for key covariates such as age, gender, depression, history of falls, comorbidities, self-rated health, life satisfaction, and physical activity. The robustness of these findings was further supported by consistent results across various subgroup analyses. From a mechanistic standpoint, pain status may interfere with both physiological and psychological processes, leading to reduced mobility, fear of movement, and elevated stress responses [24]. These disruptions can exacerbate musculoskeletal issues, impair balance, and diminish motivation to engage in daily activities, ultimately driving functional decline and disability [23]. The fear-avoidance model provides a theoretical framework to explain how pain status contributes to disability. Fear of movement, often triggered by pain, may result in avoidance behaviors, which reduce physical activity and increase the risk of functional decline. Pain-related fear has been identified as a significant predictor of self-reported disability in individuals with pain status, emphasizing the critical role of psychological factors in the progression of disability [25]. This interplay among pain, fear, and reduced activity highlights the need for targeted interventions to break the cycle of pain, fear, and inactivity, thereby mitigating the associated risk of disability.

Our findings suggest that comorbidities play a critical role in the risk of disability. While having a single chronic condition did not significantly increase disability risk, the presence of two or more conditions elevated the risk of both BADL and IADL disabilities. This may be explained by the cumulative effect of multiple comorbidities on physiological and functional capacities. Each additional chronic disease can introduce complex symptoms, treatment regimens, and limitations that interact synergistically, amplifying the overall burden on an individual's ability to perform daily activities. Previous studies have demonstrated that specific conditions, such as back pain, peripheral vascular disease, and stroke, contribute particularly to disability due to their high disabling impact [26]. Multimorbidity may reduce physical resilience and increase pain perception, thereby intensifying the functional impairment observed in older adults [27–29]. Effective management of multimorbidity in older populations is thus crucial to mitigating the risk of disability, as interventions must address not just one, but multiple interacting health challenges.

The intensity of physical activity demonstrated a more pronounced association with IADL disability than with BADL disability, highlighting the importance of maintaining moderate-to-intense activity levels to preserve complex functional abilities. This observation is consistent with the existing literature that emphasizes the protective role of physical activity against functional decline. Regular engagement in physical activity has been shown to improve muscle strength, balance, and coordination, thereby reducing the risk of disability and falls among older adults [30]. However, pain can lead to a reduction in physical activity, creating a cyclical pattern in which decreased activity worsens disability, further limiting the ability to remain active. Older adults with pain status are more prone to recurrent falls and fear of falling, factors that discourage physical activity and accelerate functional decline [31]. Encouraging regular, moderate-to-intense physical activity, along with effective pain management, is essential for reducing the risk of IADL disability and enhancing the overall quality of life in this population.

Our study also identified depressive symptoms and lower cognitive scores as significant risk factors for disability in BADL and IADL, with self-rated health exerting the greatest influence on the effect of pain on disability. The role of self-rated health was particularly pronounced, emerging as the covariate with the most substantial impact on the effect of pain on disability. The mechanism may involve the subjective nature of pain, where negative health perceptions intensify pain experiences. Older adults experiencing pain are likely to have lower self-perceptions of their health, which in turn leads to reduced life satisfaction and negatively impacts emotional well-being. The interplay among pain, self-rated health, and depression is well documented. It has been shown that depression significantly predicts disability in patients with pain [7]. Additionally, symptoms of depression and stress have been found to mediate the effect of pain on disability, further supporting the notion that psychological impact of pain is a critical factor in the progression of disability among older adults [32]. Our findings suggest that enhancing self-perceptions of health among older adults may be an effective strategy to prevent BADL- and IADL-related issues later in life. Improving self-rated health could mitigate the negative effects of pain and depression, thereby reducing the risk of disability. This is supported by research, indicating that interventions aimed at improving self-perceived health status can have a protective effect against disability [33].

Notably, our results demonstrated that multisite pain was associated with higher OR of disability compared to single-site pain. Head and neck pain specifically affected IADL disability, whereas pain in the limbs and torso influenced both BADL and IADL, indicating that the location and distribution of pain may yield distinct functional consequences. This observation is somewhat inconsistent with a study conducted in Taiwan, which reported that the IADL level, but not BADL, was significantly associated with pain [34]. However, the results of our study align with broader literature that emphasizes the detrimental effects of musculoskeletal pain on physical function. For instance, back pain is frequently identified as a major contributor to disability, particularly due to its impact on lower extremity physical function, which is crucial for mobility and daily activities [26, 35]. Furthermore, pain experienced in the shoulder, arm, wrist, back, hip, leg, and ankle has been consistently linked to BADL impairment, with multisite pain being associated with even greater levels of disability [36]. The significance of multisite pain as a risk factor for disability is increasingly recognized in clinical research and practice. As the number of pain sites increases, both the severity of pain and the extent of disability increase [37]. This cumulative effect of pain across multiple sites contributes to worse functional outcomes [30], including greater impairment in daily activities [38], increased fall risk [31], and worse health-related quality of life [39]. Additionally, multisite pain is associated with higher levels of anxiety and depression [40], further complicating the clinical management of older adults. The presence of pain in multiple locations likely imposes overlapping functional constraints, hindering a range of daily tasks and activities. This multifaceted burden underscores the need for interventions tailored to the specific pain distribution patterns in older adults. Identifying pain sites as phenotypic markers could help clinicians better predict functional trajectories and design targeted interventions [41].

## 5. Conclusion

In conclusion, this study contributes to the growing body of evidence on the associations among pain status, physical activity, depressive symptoms, comorbidities, and disability in older adults. The observed associations highlight the potential importance of addressing multisite pain, managing multimorbidity, and promoting regular moderate-to-intense physical activity in efforts to maintain functional independence. While causality cannot be inferred due to the cross-sectional nature of the data, these findings may help inform the design of more comprehensive and individualized interventions. Tailored strategies that alleviate pain, foster physical activity, and enhance psychological well-being are crucial for breaking the cycle of pain and disability. Such approaches are essential to reducing BADL and IADL disabilities, preserving functional independence, and improving the overall quality of life in aging populations. These insights underscore the need for holistic, person-centered approaches to support healthy aging.

## 6. Limitations

This study has several limitations that should be acknowledged. First, the method of pain assessment was based on self-reported responses, which, although widely used and accepted in epidemiological research [42], remains inherently subjective. The association between pain and disability may affect how individuals perceive and report their experiences—those with functional limitations may rate pain as more severe, and vice versa. Nonetheless, this potential bias in measurement is unlikely to fully account for the strong associations observed between pain status and functional disability in our study. Second, the use of self-reported BADL and IADL data may lead to under- or overestimation of disability. Although commonly used in large-scale surveys, self-reports may not fully capture actual functional capacity.

Additionally, we did not perform multiple imputation for missing data, but instead used complete-case analysis after supplementing available demographic information from earlier CHARLS waves. Participants missing key variables such as cognition and depression were excluded, as these were based on validated scales and imputation could introduce bias. The final sample size was sufficiently large to ensure statistical robustness. Future research should incorporate objective assessments and longitudinal data to further explore the temporal and causal relationships involved.

## Figures and Tables

**Table 1:** Baseline characteristics of the study participants.

	Total	No pain	Pain	*p* value
	*N* = 8102	*N* = 3343	*N* = 4759	
Age	68.00 [64.00; 73.00]	68.00 [64.00; 73.00]	68.00 [64.00; 73.00]	0.396
Gender, *n* (%)				< 0.001
Female	4030 (49.74)	1315 (39.34)	2715 (57.05)	
Male	4072 (50.26)	2028 (60.66)	2044 (42.95)	
Marital status, *n* (%)				0.001
Unmarried	6229 (76.88)	2635 (78.82)	3594 (75.52)	
Married	1873 (23.12)	708 (21.18)	1165 (24.48)	
Social participation, *n* (%)				< 0.001
No	4255 (52.52)	1835 (54.89)	2420 (50.85)	
Yes	3847 (47.48)	1508 (45.11)	2339 (49.15)	
Education, *n* (%)				< 0.001
No education	1995 (24.62)	698 (20.88)	1297 (27.25)	
Primary school	3504 (43.25)	1402 (41.94)	2102 (44.17)	
Middle school	1559 (19.24)	729 (21.81)	830 (17.44)	
High school/above	1044 (12.89)	514 (15.38)	530 (11.14)	
Living area, *n* (%)				< 0.001
Urban	5328 (65.76)	2103 (62.91)	3225 (67.77)	
Rural	2774 (34.24)	1240 (37.09)	1534 (32.23)	
Smoking, *n* (%)				1.000
No	4371 (97.87)	1550 (97.85)	2821 (97.88)	
Yes	95 (2.13)	34 (2.15)	61 (2.12)	
Drinking, *n* (%)				< 0.001
No	5264 (64.97)	2016 (60.31)	3248 (68.25)	
Yes	2838 (35.03)	1327 (39.69)	1511 (31.75)	
Falls, *n* (%)				< 0.001
No	6677 (82.41)	3010 (90.04)	3667 (77.05)	
Yes	1425 (17.59)	333 (9.96)	1092 (22.95)	
Self-report health, *n* (%)				< 0.001
Good	1754 (21.65)	1215 (36.34)	539 (11.33)	
Fair	4160 (51.35)	1731 (51.78)	2429 (51.04)	
Poor	2188 (27.01)	397 (11.88)	1791 (37.63)	
Life satisfaction, *n* (%)				< 0.001
Completely	3006 (37.10)	1493 (44.66)	1513 (31.79)	
Somewhat	4324 (53.37)	1681 (50.28)	2643 (55.54)	
Not satisfied	772 (9.53)	169 (5.06)	603 (12.67)	
Physical activity, *n* (%)				0.675
Light	3723 (45.95)	1555 (46.52)	2168 (45.56)	
Moderate	3995 (49.31)	1629 (48.73)	2366 (49.72)	
Intensive	384 (4.74)	159 (4.76)	225 (4.73)	
Cognition	20.00 [16.00; 24.00]	20.00 [16.00; 24.00]	20.00 [16.00; 23.00]	0.003
Depression, *n* (%)				< 0.001
No	5171 (63.82)	2625 (78.52)	2546 (53.50)	
Yes	2931 (36.18)	718 (21.48)	2213 (46.50)	
Comorbidities, *n* (%)				< 0.001
0	4967 (61.31)	2335 (69.85)	2632 (55.31)	
1	2037 (25.14)	733 (21.93)	1304 (27.40)	
≥ 2	1098 (13.55)	275 (8.23)	823 (17.29)	
BADL disability, *n* (%)				< 0.001
No	6026 (74.38)	2965 (88.69)	3061 (64.32)	
Yes	2076 (25.62)	378 (11.31)	1698 (35.68)	
IADL disability, *n* (%)				< 0.001
No	5934 (73.24)	2880 (86.15)	3054 (64.17)	
Yes	2168 (26.76)	463 (13.85)	1705 (35.83)	

**Table 2:** Multivariate logistic regression for factors affecting BADL and IADL.

	BADL		IADL		VIF
	OR (95% CI)	*p* value	OR (95% CI)	*p* value	
Pain					1.06
No	Reference		Reference		
Yes	2.34 (2.04–2.69)	< 0.001	1.91 (1.67–2.19)	< 0.001	
Age	1.04 (1.03–1.05)	< 0.001	1.06 (1.05–1.07)	< 0.001	1.03
Gender					1.09
Female	Reference		Reference		
Male	0.84 (0.74–0.95)	0.006	0.75 (0.67–0.85)	< 0.001	
Education					1.17
No education	Reference		Reference		
Primary school	0.8 (0.69–0.92)	0.003	0.85 (0.74–0.99)	0.034	
Middle school	0.74 (0.61–0.9)	0.003	0.86 (0.71–1.05)	0.130	
High school/above	0.66 (0.52–0.84)	0.001	0.63 (0.49–0.8)	< 0.001	
Depression					1.10
No	Reference		Reference		
Yes	1.76 (1.56–2)	< 0.001	1.96 (1.74–2.22)	< 0.001	
Comorbidities					1.01
0	Reference		Reference		
1	1.08 (0.95–1.24)	0.244	1.09 (0.95–1.25)	0.202	
≥ 2	1.35 (1.15–1.58)	< 0.001	1.30 (1.11–1.52)	0.001	
Self-report health					1.04
Good	Reference		Reference		
Fair	1.75 (1.44–2.12)	< 0.001	1.5 (1.25–1.8)	< 0.001	
Poor	4.26 (3.49–5.24)	< 0.001	4.31 (3.55–5.25)	< 0.001	
Life satisfaction					1.04
Completely	Reference		Reference		
Somewhat	1.05 (0.93–1.2)	0.424	1.04 (0.91–1.18)	0.551	
Not satisfied	1.68 (1.38–2.05)	< 0.001	1.62 (1.33–1.97)	< 0.001	
Physical activity					1.01
Light	Reference		Reference		
Moderate	0.9 (0.8–1.01)	0.066	0.86 (0.76–0.97)	0.011	
Intensive	0.75 (0.56–1)	0.054	0.64 (0.47–0.85)	0.003	
Falls					1.01
No	Reference		Reference		
Yes	2.27 (1.98–2.59)	< 0.001	1.57 (1.37–1.8)	< 0.001	
Cognition	0.99 (0.97–1)	0.029	0.96 (0.95–0.97)	< 0.001	1.17

Abbreviations: CI = confidence interval; OR = odds ratio; VIF = variance inflation factor.

**Table 3:** Impact of pain location and pain sites on BADL and IADL disability.

	BADL				IADL			
	Unadjusted		Adjusted		Unadjusted		Adjusted	
	OR (95% CI)	*p* value	OR (95% CI)	*p*value	OR (95% CI)	*p* value	OR (95% CI)	*p* value
Head and neck	2.91 (2.62–3.24)	< 0.001	1.00 (0.87–1.16)	0.965	2.92 (2.63–3.25)	< 0.001	1.22 (1.06–1.41)	0.006
Upper limb	3.33 (3.00–3.70)	< 0.001	1.25 (1.08–1.45)	0.003	2.85 (2.57–3.16)	< 0.001	1.25 (1.08–1.45)	0.003
Torso	3.48 (3.13–3.86)	< 0.001	1.25 (1.09–1.44)	0.002	3.01 (2.72–3.33)	< 0.001	1.27 (1.1–1.46)	0.001
Lower limb	4.65 (4.18–5.18)	< 0.001	2.26 (1.96–2.61)	< 0.001	3.19 (2.88–3.53)	< 0.001	1.38 (1.2–1.59)	< 0.001
Pain distribution								
No pain	Reference		Reference		Reference		Reference	
Single site	1.88 (1.56–2.27)	< 0.001	1.39 (1.14–1.70)	0.001	1.77 (1.49–2.11)	< 0.001	1.35 (1.12–1.64)	0.002
Multisite	5.31 (4.68–6.02)	< 0.001	2.72 (2.36–3.14)	< 0.001	4.10 (3.64–4.62)	< 0.001	2.13 (1.85–2.45)	< 0.001

Abbreviations: CI = confidence interval; OR = odds ratio.

**Table 4:** Odds ratios for pain-related disability risk across demographic and health-related subgroups.

Variable	Count (*N*)	Percent (%)	BADL		IADL	
			OR (95% CI)	*p* value	OR (95% CI)	*p* value
Overall	8102	100.00	2.34 (2.04–2.69)	< 0.001	1.91 (1.67–2.19)	< 0.001
Age						
< 70	4829	59.60	2.60 (2.14–3.15)	< 0.001	1.74 (1.45–2.10)	< 0.001
≥ 70	3273	40.40	2.10 (1.72–2.57)	< 0.001	2.01 (1.66–2.43)	< 0.001
Gender						
Female	4030	49.74	2.53 (2.07–3.08)	< 0.001	1.82 (1.51–2.19)	< 0.001
Male	4072	50.26	2.19 (1.81–2.65)	< 0.001	1.90 (1.57–2.29)	< 0.001
Education						
No education	1995	24.62	2.27 (1.75–2.95)	< 0.001	1.81 (1.41–2.33)	< 0.001
Primary school	3504	43.25	2.82 (2.28–3.49)	< 0.001	1.91 (1.57–2.34)	< 0.001
Middle school	1559	19.24	1.82 (1.32–2.50)	< 0.001	1.76 (1.29–2.40)	< 0.001
High school/above	1044	12.89	2.43 (1.59–3.74)	< 0.001	2.22 (1.38–3.56)	< 0.001
Health						
Good	1754	21.65	3.77 (2.60–5.45)	< 0.001	2.55 (1.81–3.60)	< 0.001
Fair	4160	51.35	2.26 (1.88–2.71)	< 0.001	1.90 (1.58–2.27)	< 0.001
Poor	2188	27.01	2.01 (1.57–2.57)	< 0.001	1.63 (1.28–2.08)	< 0.001
Depression						
No	5171	63.82	2.31 (1.94–2.76)	< 0.001	1.89 (1.59–2.25)	< 0.001
Yes	2931	36.18	2.41 (1.93–3.00)	< 0.001	1.86 (1.52–2.29)	< 0.001
Comorbidities						
0	4967	61.31	2.50 (2.09–2.99)	< 0.001	1.96 (1.65–2.32)	< 0.001
1	2037	25.14	2.12 (1.63–2.77)	< 0.001	1.75 (1.35–2.27)	< 0.001
≥ 2	1098	13.55	2.21 (1.52–3.22)	< 0.001	1.66 (1.15–2.41)	< 0.001

Abbreviations: CI = confidence interval; OR = odds ratio.

## Data Availability

The data that support the findings of this study are openly available in China Health and Retirement Longitudinal Study, CHARLS at https://charls.pku.edu.cn/, reference number doi: 10.1093/ije/dys203.
